# 3D Reconstruction of *Hv*RNASET2 Molecule to Understand Its Antibacterial Role

**DOI:** 10.3390/ijms21249722

**Published:** 2020-12-19

**Authors:** Nicolò Baranzini, Laura Pulze, Marcella Reguzzoni, Rossella Roncoroni, Viviana Teresa Orlandi, Gianluca Tettamanti, Francesco Acquati, Annalisa Grimaldi

**Affiliations:** 1 Department of Biotechnology and Life Science, University of Insubria, 21100 Varese, Italy; n.baranzini@uninsubira.it (N.B.); l.pulze@uninsubria.it (L.P.); rroncoroni@studenti.uninsubria.it (R.R.); viviana.orlandi@uninsubria.it (V.T.O.); gianluca.tettamanti@uninsubria.it (G.T.); 2 Department of Medicine and Surgery, University of Insubria, 21100 Varese, Italy; marcella.reguzzoni@uninsubria.it

**Keywords:** 3D reconstruction of *Hv*RNASET2, antimicrobial activity, LPS, innate immunity, medicinal leech

## Abstract

Recent studies performed on the invertebrate model *Hirudo verbana* (medicinal leech) suggest that the T2 ribonucleic enzyme *Hv*RNASET2 modulates the leech’s innate immune response, promoting microbial agglutination and supporting phagocytic cells recruitment in challenged tissues. Indeed, following injection of both lipoteichoic acid (LTA) and *Staphylococcus aureus* in the leech body wall, *Hv*RNASET2 is expressed by leech type I granulocytes and induces bacterial aggregation to aid macrophage phagocytosis. Here, we investigate the *Hv*RNASET2 antimicrobial role, in particular assessing the effects on the Gram-negative bacteria *Escherichia coli*. For this purpose, starting from the three-dimensional molecule reconstruction and in silico analyses, the antibacterial activity was evaluated both in vitro and in vivo. The changes induced in treated bacteria, such as agglutination and alteration in wall integrity, were observed by means of light, transmission and scanning electron microscopy. Moreover, immunogold, AMPs (antimicrobial peptides) and lipopolysaccharide (LPS) binding assays were carried out to evaluate *Hv*RNASET2 interaction with the microbial envelopes and the ensuing ability to affect microbial viability. Finally, in vivo experiments confirmed that *Hv*RNASET2 promotes a more rapid phagocytosis of bacterial aggregates by macrophages, representing a novel molecule for counteracting pathogen infections and developing alternative solutions to improve human health.

## 1. Introduction

Bacterial infections represent one of the major problems for human health, causing different types of diseases. In the last century, although the discovery and use of antibiotics have improved the possibility to counteract harmful microorganisms, their poorly controlled and inappropriate application has led to an effective bacterial resistance against these molecules, based on various mechanisms of defense, in order to avoid antibiotic effects [1]. In this context, hospital nosocomial infections constitute an ongoing serious danger, especially for immunocompromised individuals or those with critical illnesses, of which different pathogenic bacteria often represent the main primary cause due to their ability to prevent any potential drug effect. Despite the development of novel antibiotic therapies still representing an efficient method, this approach not only presents an elevated cost but also risks causing further pathogen resistance [2]. Thus, in order to improve the research of innovative strategies, in particular in clinical systems, both the study of alternative antimicrobial molecules and the identification of new models appear necessary. In recent decades, the discovery of many antimicrobial peptides opened the door to the possibility to effectively design new therapeutic tools. However, although these molecules show both high validity and selectivity for different pathogens, their cytotoxicity against host cells often indicates a disadvantage of their possible use [3]. 

In the investigation of potential solutions, it has been observed that many enzymes are efficiently active against different harmful agents, and their application has been widely examined. Among these enzymes, ribonucleases recently acquired a certain importance due to their ability to take part in the regulation of several biological processes, including immune response. In particular, enzymes belonging to the T2 ribonuclease family (T2 RNases), representing a widespread group of proteins present in almost all organisms and possessing a highly conserved RNAse-regulating housekeeping role, are also involved in several key cellular processes [4]. Indeed, by showing a high pleiotropic nature, T2 RNases have apparently acquired specific functionalities during their evolution in different organisms [5,6].

Interestingly, the involvement of T2 RNases in immune processes has been already demonstrated in plants, in which several ribonucleases are implicated in the defensive mechanisms of seeds and branches. Indeed, these proteins are able to counteract external harmful agents, such as viruses or bacteria, independently from the catalytic activity [4,6]. In humans, overexpression of the RNASET2 enzyme regulates the immune response in mice carcinoma models, promoting a pro-inflammatory response, recruiting immunocompetent cells and inducing M1/M2 macrophage phenotype transition in the microenvironment surrounding the tumor mass, which in turns triggers a marked oncosuppressive activity [7,8]. Interestingly, the ability to chemoattract macrophages has also been observed in the medicinal leech *H. verbana*, in which the T2 RNase enzyme *Hv*RNASET2 appears directly involved in the modulation of innate immunity [9,10]. 

As observed in vertebrate models, the injection in the leech body wall of LPS and LTA, the main antigens of Gram-negative and Gram-positive, respectively, triggered an increased expression of the endogenous *Hv*RNASET2 in both host type I granulocytes and macrophages, indicating a direct modulating role of the innate immune response during bacterial infections [9,10]. In *H. verbana*, type I granulocytes represent one of the first cellular populations activated during bacterial challenges. Once stimulated, these cells degranulate the content of their cytoplasmatic granules in the extracellular environment, releasing different types of immune molecules [11]. When released, *Hv*RNASET2 agglutinated Gram-positive bacteria *Staphylococcus aureus* both in vivo and in vitro, also altering the cell integrity. Indeed, the close interaction with the microorganism cell walls was confirmed by its specific localization on the bacterial external surfaces [10]. The *Hv*RNASET2 expression pattern during the earlier phases of leech inflammation, coupled with the simultaneous burst of macrophages recruitment, confirmed its crucial role in counteracting pathogens [9]. In particular, the simultaneous chemoattractant role of *Hv*RNASET2 against macrophages and the formation of bacterial clusters seem to be critically involved not only in increasing the number of pathogen-targeting immune cells but also in improving the macrophage phagocytic activity itself, a key prerequisite for the elimination of pathogens and possible cellular debris [10]. These results suggest that T2 RNases represent evolutionarily conserved key molecules implicated in the regulation of pathogen–host defense processes [10,12], whose role should be better investigated in the search for new solutions against different types of infections.

Based on this evidence and on the results previously achieved with *S. aureus*, in the present study, we assessed the *Hv*RNASET2 effects on the Gram-negative bacteria *E. coli*. 3D structure analysis has been predicted to further confirm the high level of conservation existing between T2 RNase family members. Furthermore, *Hv*RNASET2 bioinformatics characterization was crucial not only for demonstrating the preservation of conserved peptide motifs, but also to identify particular amino acid residues that might explain the obtained results. Subsequently, the agglutinating ability of the leech RNase T2 enzyme was evaluated both in vitro and in vivo, revealing that it indifferently recognizes any type of bacterium, affecting bacterial viability and promoting macrophages phagocytosis.

## 2. Results

### 2.1. Bioinformatic Characterization of H. verbana HvRNASET2

**Figure 1:**
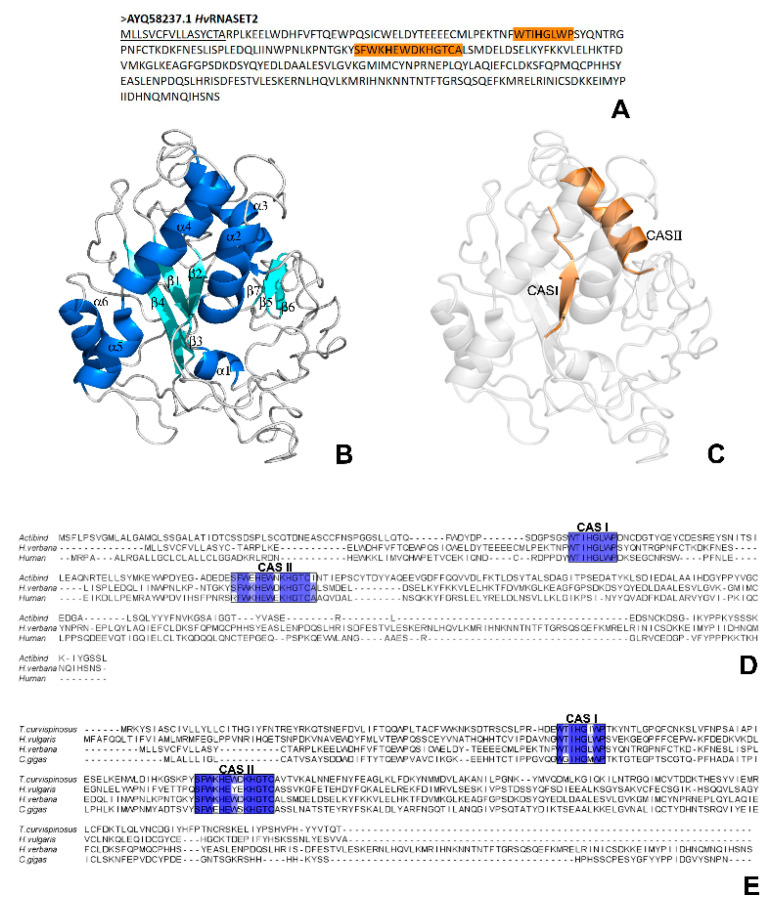
*Hv*RNASET2 bioinformatic characterization. (**A**) *Hv*RNASET2 (accession number: AYQ58237.1) amino acid sequence. In the N-terminal region, the underlined residues identify the signal peptide, and the two conserved active sites (CAS) are underscored in bright orange. (**B**,**C**) Secondary HvRNASET2 three-dimensional structure, predicted with I-TASSER. The β-strands and α-helix that composed the secondary structure are represented in cyan and blue (**B**). The CAS I and CAS II domains, reported in bright orange (**C**), are specifically located on the β2-strand and α3-helix, respectively. (**D**,**E**) Multiple sequence alignments. The *H. verbana Hv*RNASET2 amino acid sequence was aligned either with those of the well-studied *A. niger* ACTIBIND and human RNASET2 enzymes (**D**) or with *T. curvispinus* (Arthropoda), *H. vulgaris* (Cnidaria) and *C. gigas* (Mollusca) (**E**) invertebrate T2 RNases. Both the CAS I and CAS II domains are extremely well conserved, and different blue shades identify the distinct conservation of each specific amino acid.

The main bioinformatics parameters of *Hv*RNASET2 (accession number: AYQ58237.1) were investigated using different servers and software. Analysis of the primary sequence (Figure 1A) revealed that the leech T2 enzyme consisted of 304 amino acids, with a predicted molecular weight of 35.8 kDa, as expected by previous results [12]. The PROSITE server confirmed the presence of the two specific conserved active sites, CAS I and CAS II, which are involved in the housekeeping catalytic activity and are extremely well conserved in Rh/T2/S ribonucleases. These active domains were specifically observed in positions Trp60-Pro67 (WTIHGLWP) and Ser110-Ala123 (SFWKHEWDKHGTCA), respectively (Figure 1A). Moreover, the two crucial histidine residues (His61 and His117) were detected in the two active sites. The analysis conducted with SignalP-5.0 revealed the presence of an N-terminal signal peptide (Figure 1A) of sixteen amino acids (MLLSVCFVLLASYCTA), proving that *Hv*RNASET2 is normally secreted toward the secretory pathway, as also confirmed by the TMHMM server that identified its extracellular position.

The *Hv*RNASET2 three-dimensional structure, predicted by I-TASSER, was subsequently analyzed with the PyMOL software in order to better visualize the secondary structures. In particular, the typical α + β motif of T2 RNases were observed in *Hv*RNASET2 as well, with the central core of β-sheet, flanked by a close helical region (Figure 1B). In particular, the two highly conserved residues CAS I and CAS II were respectively localized on the central β2-strand and on the relative parallel α3-helix (Figure 1C), in which the two specific histidine residues were also present, as demonstrated for many T2 RNase members. In order to evaluate the quality of the model predicted with I-TASSER, a three-dimensional alignment was performed between *Hv*RNASET2 and human RNASET2 (PDB code: 3T0O) (Figure S1A), and the QMEANDisCo values of each amino acid were graphically represented (Figure S1B). To further demonstrate the high evolutionary conservation, multiple sequence alignments were conducted using the Clustal Omega program, by which the *Hv*RNASET2 amino acid sequence was compared with that of other T2 RNases. The *H. verbana* T2 enzyme was aligned both with *Aspergillus niger* ACTIBIND and human RNASET2 (Figure 1D) and with the T2 ribonucleases from the following invertebrate organisms: *Hydra vulgaris* (Cnidaria), *Crassostrea gigas* (Mollusca) and *Temnothorax curvispinosus* (Arthropoda) (Figure 1E). The obtained results revealed that the two CAS I and CAS II domains were highly conserved in all the analyzed sequences (Figure 1D,E), as also demonstrated by the complete alignment of all amino acid sequences (Figure S1F). 

**Figure 2:**
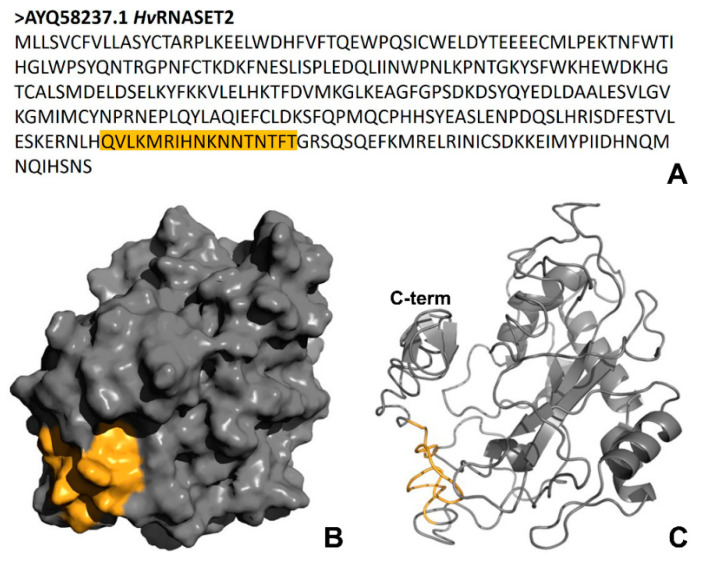
Antimicrobial peptide identification. (**A**) The HvRNASET2 primary sequence was analyzed with the AMPA software [13] in order to identify possible antimicrobial peptides, and a specific sequence of amino acids (QVLKMRIHNKNNTNTFT), underscored in orange, was detected in the C-terminal region. (**B**,**C**) Three-dimensional representations of the detected peptide are displayed in orange. Of note, it is arranged on the outer surface (**B**) and in the C-terminal region (NQMNQIHSNS) (**C**) of the enzyme.

Based on recent evidence in which the *Hv*RNASET2 antibacterial role was observed by both in vivo and in vitro experiments [10], the bioinformatic characterization was also focused on the theoretical identification of any possible antimicrobial peptides (Figure 2). By using the AMPA software algorithm, which predicted possible antimicrobial stretches in a protein’s primary sequence, we detected a specific domain in the position Gln247-Thr263 presenting an encouraging probability score (Figure 2A). Interestingly, the three-dimension display of the enzyme surface showed that this amino acid sequence was externally located (Figure 2B) and placed in a region of disorder of the C-terminal region (QVLKMRIHNKNNTNTFT) (Figure 2C). The precise localization of the peptide on the *Hv*RNASET2 surface was also confirmed by the construction of several models using different software (Figure S1C–E). 

### 2.2. In Vitro Evaluation of the HvRNASET2 Antibacterial Role

**Figure 3:**
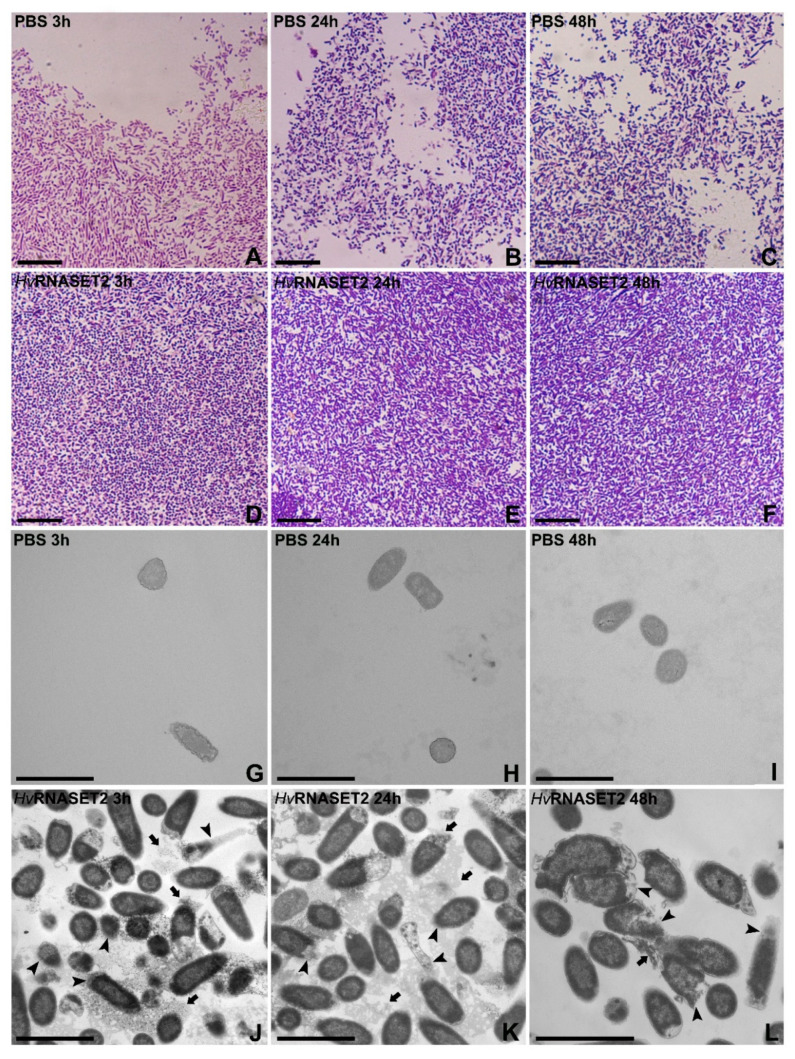
Optical and TEM morphological analyses. (**A**–**F**) *E. coli* incubated with PBS (**A**–**C**) or rHvRNASET2 (**D**–**F**) were assessed by optical microscope after 3 (**A**,**D**), 24 (**B**,**E**) and 48 h (**C**,**F**) from the incubation. The bacterial distribution was dense, and microorganisms appeared closer after r*Hv*RNASET2 treatment (**D**–**F**) compared to PBS control samples (**A**–**C**). (**G**–**L**) *E. coli* incubated with PBS (**G**–**I**) or r*Hv*RNASET2 (**J**–**L**) analyzed with TEM. After 3 (**G**,**J**), 24 (**H**,**K**) and 48 h (**I**,**L**) from incubation, in control samples, cells maintained their typical shape (**G**–**I**), while after incubation with r*Hv*RNASET2, they were clearly agglutinated and suffering (**J**–**L**). Indeed, bacterial membranes appear damaged (arrowheads), and the cytoplasmatic content was released in the surrounded environment (arrows). Scale bars: (**A**–**F**) 10 µm; (**G**–**L**) 2 µm.

The *Hv*RNASET2 ability to directly affect microorganisms was tested in vitro on a Gram-negative *E. coli* bacterial strain (Figure 3). Morphological analyses were performed after living-cell incubation with the recombinant enzyme, expressed and produced by X33 *Pichia pastoris* strain [14], observing the effects after 3, 24 and 48 h (Figure 3A–F). Images obtained with the light microscope showed that, unlike PBS-treated control samples, in which bacteria appeared randomly distributed (Figure 3A–C), r*Hv*RNASET2 treatment induced an evident bacterial agglutination at all time points (Figure 3D–F). The same effects were better detected at TEM (Figure 3G–L), in which microorganisms incubated with PBS were randomly distributed (Figure 3G–I), whereas recombinant r*Hv*RNASET2-treated bacteria appeared closer (Figure 3J–L). Moreover, TEM details showed that *Hv*RNASET2-treated bacteria showed damaged cell membranes, and their cytoplasmatic content was released in the surrounding medium, indicating the destabilizing effect of *Hv*RNASET2 on them.

### 2.3. HvRNASET2 Interaction with E. coli

**Figure 4:**
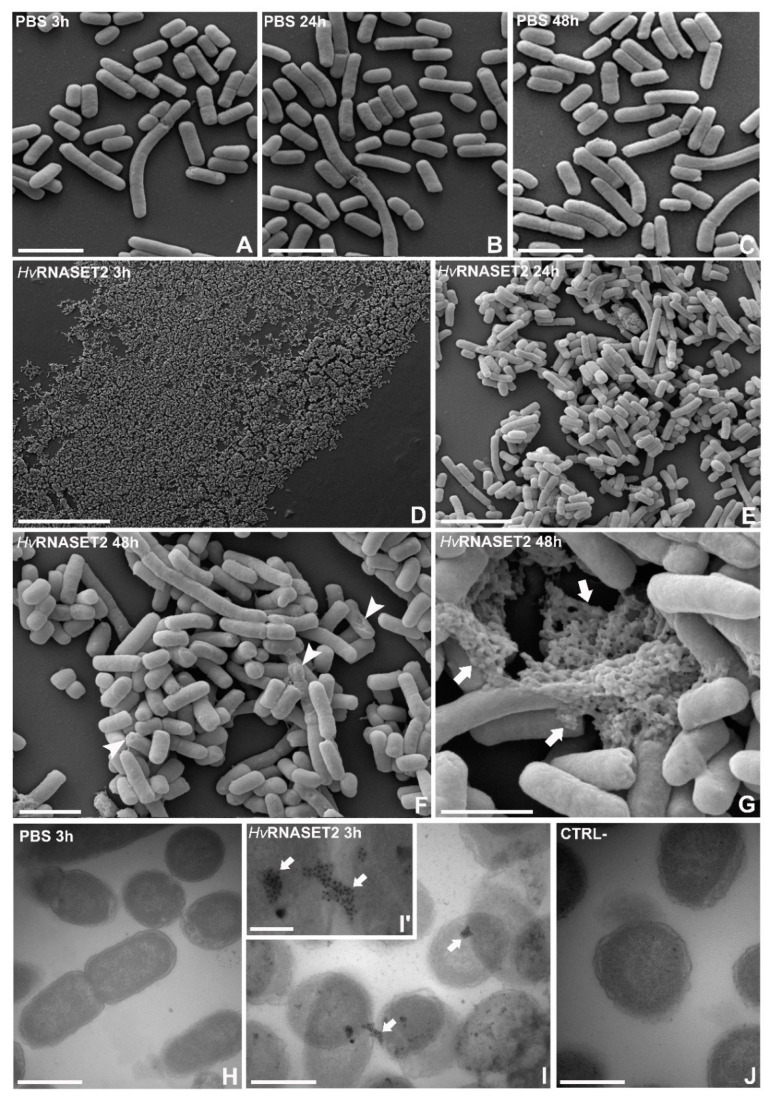
SEM and immunogold analyses. (**A**–**C**) With SEM, *E. coli* treated with PBS were randomly distributed on the analyzed surfaces, after 3 h (**A**), 24 h (**B**) and 48 h (**C**). (**D**–**G**) However, the incubation with rHvRNASET2 induced a strong bacterial agglutination already 3 h after treatment (**D**). Moreover, in particular after 24 h (**E**) and 48 h (**F**,**G**) from incubation, bacterial aggregates were clearly visible, and cell membranes appeared damaged (**F**) (arrowheads), releasing the cytoplasmatic content into the external environment (**G**) (arrows). (**H**–**J**) Immunogold at TEM. Immunogold assays, performed 3 h after incubation, indicate that rHvRNASET2 is specifically located on the *E. coli* outer bacterial surface (**I**,**I’**) (arrowheads). No signals were detected in PBS samples (**H**) or in negative control experiments (**J**), in which the primary antibody was omitted. In particular (**I’**), many gold particles were detectable, suggesting the direct interaction of *Hv*RNASET2 with bacterial cell components (arrow). Scale bars: (**A**–**C**,**F**) 2 µm; (**D**) 50 µm; (**E**) 5 µm; (**G**) 1 µm; (**H–J**) 0.5 µm; (**I’**) 100 nm.

The antibacterial role of *Hv*RNASET2 was also evaluated by SEM analyses (Figure 4). *E. coli* cells treated with PBS (Figure 4A–C) were randomly diffused on the examined surface, both after 3, 24 and 48 h from incubation, without evident morphological changes. In contrast, the same cells appeared highly agglutinated after *Hv*RNASET2 incubation at all time points (Figure 4D–G). Especially after 24 and 48 h, several cells displayed apparently damaged membranes, presenting an irregular shape (Figure 4F,G). Moreover, the presence of cytoplasmic material deposited between bacterial cells confirmed the ability of the leech T2 enzyme to destabilize the bacterial membranes, leading to the release of the cell content (Figure 4G) [15].

To observe the possible interaction with the bacterial outer membranes, immunogold assays with TEM were performed using a specific anti-RNASET2 primary antibody after 3 h from incubation. TEM images revealed the presence of several gold particles localized on Gram-negative bacterial surfaces, indicating the direct interaction of *Hv*RNASET2 with outer membranes of the microorganisms (Figure 4I,I’). In control experiments, in which samples were treated with PBS (Figure 4H) or in which the primary antibody was omitted (Figure 4J), no signals were detected. The fluorescent binding assay highlights that *Hv*RNASET2 is able to bind the Gram-negative antigen LPS with a higher affinity in comparison to Polymixin B (PXB), a powerful LPS binder, which is thus used as a positive control [16]. 

### 2.4. Antimicrobial Activity Assay

**Figure 5:**
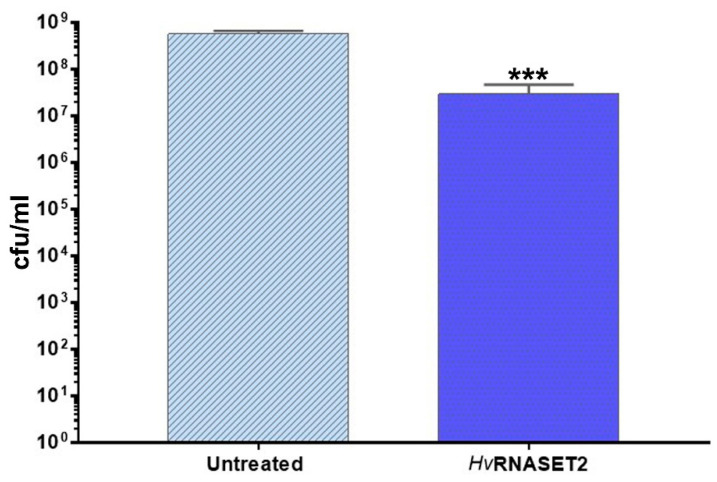
Antimicrobial in vitro assay. The graph illustrates the cellular viability of *E. coli* after 24 h of r*Hv*RNASET2 treatment. Untreated cells were used as control. Experiments were performed in triplicate, and statistical differences were calculated by unpaired *t*-test. ***p < 0.001 indicates a significant difference with untreated samples.

The effect of r*Hv*RNASET2 on *E. coli* growth was tested in vitro. The administration of the purified enzyme to active metabolizing cells affected in a significant manner (*p* = 3.8 × 10^−5^) the cell growth of *E. coli*. As can be observed in Figure 5, upon 24 h of incubation, the cellular concentration of treated samples showed a decrease of ~1 log unit compared with the untreated controls (Figure 5). 

### 2.5. In Vivo Evaluation of the Antibacterial Role 

**Figure 6:**
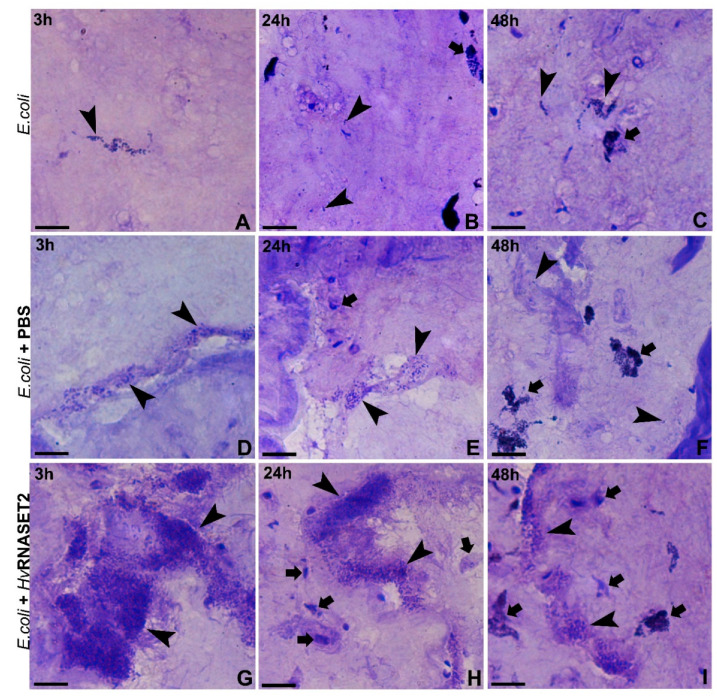
In vivo experiments. (**A**–**C**) *E. coli* alone injected in the leech body wall. Tissues were analyzed 3 h after treatment; bacterial cells appeared randomly distributed (arrowheads) and only a few resident cells (arrows) were detectable. (**D**–**F**) *E. coli* were pre-incubated with PBS for 3, 24 and 48 h and injected into the leech body wall. Tissues were analyzed after 3 h of treatment, and microorganisms appeared randomly distributed (arrowheads) with few resident cells clearly visible (arrows). (**G**–**I**) *E. coli* pre-incubated with r*Hv*RNASET2 for 3, 24 and 48 h and subsequently injected into the leech body wall. Tissues were analyzed 3 h after treatment, and bacterial cells appeared extremely agglutinated. The microorganism aggregates were evident (arrowheads) for all times, and a larger number of endogenous leech cells were observed surrounding bacterial clusters (arrows). These data confirm the ability of *Hv*RNASET2 in recruiting immune cells to the challenged area. Scale bars: (**A**–**I**) 10 µm.

The ability of *Hv*RNASET2 to both affect possible pathogens viability and regulate the leech innate immune system was evaluated by in vivo experiments (Figure 6A–I). Unlike control samples, in which microorganisms were injected either alone (Figure 6A–C) or pre-incubated with PBS (Figure 6D–F) for 3, 24 and 48 h, treatment with r*Hv*RNASET2 (Figure 6E–G) induced a massive bacterial agglutination. Indeed, *E. coli* control cells appeared randomly dispersed in the analyzed tissues, and only a few endogenous leech immune cells were clearly detectable (Figure 6A–F). However, *Hv*RNASET2 not only induced the formation of various bacterial clumps already 3 h after incubation (Figure 6G), but also allowed the recruitment of many cells in order to counteract and eliminate the injected cells (Figure 6G,I). As already observed [10], *Hv*RNASET2 thus displayed a double role, agglutinating microorganisms and favoring both phagocytic and immune defensive events. 

**Figure 7:**
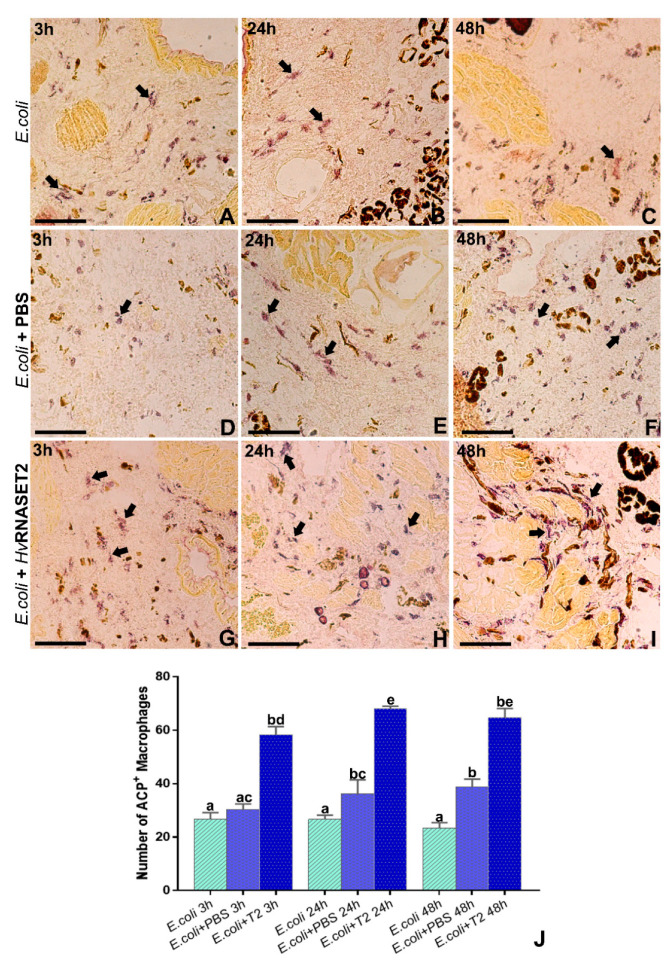
ACP assay. (**A**–**C**) *E. coli* alone injected in the leech body wall and tissues were analyzed after 3 h from treatment. Several ACP^+^-activated macrophages were detected in the challenged area. These cells are recognizable by the intense cytoplasmatic lysosomial activity (arrows). (**D**–**F**) *E. coli* were pre-incubated with PBS for 3 h, 24 h and 48 h and injected into the leech body wall. Tissues were analyzed 3 h after treatment, and the number of ACP^+^ presented cells (arrows) was quite similar to those observed when bacteria are injected alone. (**G**–**I**) *E. coli* were pre-incubated with r*Hv*RNASET2 for 3 h, 24 h and 48 h and subsequently injected into the leech body wall. Tissues were analyzed after 3 h from treatment, and many ACP^+^ macrophages are detected (arrows). (**J**) Graph showing the total positive ACP cells count. Means with different letters indicate a significant difference between treatments at different times. Experiments were performed in triplicate, and data represent mean values ± SD. Statistical analyses were performed using Statistica 7.0 software, and differences were calculated by one-way ANOVA followed by Tukey’s post hoc test. *p* < 0.05 was considered statistically significant. Scale bars: (**A**–**I**) 100 µm. Different letters indicate statistically significant differences (*p* < 0.05).

In order to confirm the recruitment and the activation of phagocytic macrophages, acid phosphatase assays were performed (Figure 7). After the injection of *E. coli* alone (Figure 7A–C) or pre-incubated with PBS (Figure 7D–F) or with r*Hv*RNASET2 (Figure 7G–I), several activated ACP^+^ macrophages were recruited. However, as also shown in the graph (Figure 7J), which also represents the number of activated cells, the total of ACP^+^ phagocytic macrophages appeared much higher when bacteria were pretreated with the leech T2 RNase. These data confirmed the ability of *Hv*RNASET2 to trigger macrophages in the activated area [9] compared with control samples.

## 3. Discussion

The emergence of novel antimicrobial agents to counteract possible harmful agents is derived from the necessity to adopt alternative and innovative therapeutic solutions. Indeed, the capacity of many bacterial strains to acquire multi-drug resistance has made most common treatments ineffective, causing an increase in problems both for public health and the economic status of several national health programs, thus stimulating the search for innovative molecules to be used as alternative powerful tools to replace the expensive and poorly effective treatments already used [17,18]. 

In this context, it has long been known that many enzymes are involved in host defense against different pathogens, affecting microorganisms’ viability and modulating the immune response. Among them, it was recently demonstrated in the medicinal leech H. verbana that the HvRNASET2 enzyme plays a pivotal role in host defense, especially during bacterial infections. Indeed, injection in the leech body wall of both the bacterial antigens LPS and LTA induces a rapid increase in the endogenous expression of this enzyme [9,10]. Moreover, in vivo and in vitro experiments carried out on Gram-positive S. aureus cells revealed that HvRNASET2 agglutinates living cells and triggers the formation of several bacterial clusters. In addition, several blebs were clearly visible on cell membranes, indicating a local destabilization [10]. 

Based on these data, in the present study, the antibacterial role of *Hv*RNASET2 has been better evaluated by means of in silico, in vitro and in vivo analyses. In particular, we assessed its ability to affect Gram-negative bacteria, in particular the *E. coli* BL21(DE3) strain, revealing how *Hv*RNASET2 actually interacts independently with different types of microorganisms. 

A first bioinformatic characterization revealed that the *H. verbana* T2 ribonuclease presents a high level of conservation with other members of the T2 RNase family and, by analyzing both the primary amino acid sequence and the three-dimensional secondary structure, many similarities were clearly detected. Indeed, the presence of the two conserved Rh/T2/S ribonuclease-specific active sites, CAS I and CAS II, was confirmed by the PROSITE server. These fundamental domains, respectively localized in positions Trp60-Pro67 and Ser110-Ala123 in the HvRNASET2 protein, are normally involved in the enzymatic housekeeping role of T2 RNases, which consists in the control of RNA processing or degradation. In particular, the three-dimensional conformation predicted with I-TASSER not only confirmed a typical α + β structural organization, with a central core of β-sheets surrounded by several α-helices, but also identified the precise localization of the CAS I and CAS II domains, respectively, on the central β2-strand and on the parallel α3-helix, as for other RNase T2 enzymes [19,20,21]. The functional relevance of these putative catalytic sites was further confirmed by the presence of the two His residues (His61 and His117), which were critically involved in the catalytic activity in both active sites [22,23]. This evidence was also validated by multiple sequence alignments to compare the leech HvRNASET2 sequence with those from different T2 RNases. In particular, the alignment with both the human RNASET2 and A. niger ACTIBIND or with several invertebrate T2 members highlighted the current similarities. 

Of note, bioinformatic analyses carried out with the AMPA software, which predicts the presence of putative antimicrobial peptides in a protein of interest [13], showed the existence of an amino acid motif with potential antibacterial properties in the *Hv*RNASET2 C-terminal region. By evaluating specific parameters, it was observed that this domain possesses both an elevated Boman index (3.04 kcal/mol) and a positive total net charge (+3.25). Interestingly, the Boman index indicates the probability for a peptide to directly bind to biological membranes, while the presence of a positive charge suggests a mechanism of interaction similar to that observed for many AMPs. Indeed, antimicrobial peptides are cationic and amphipathic molecules, which interact with the negative charges of the external bacterial walls in order to trigger membrane disruption and the activation of intracellular processes that lead to the death of microorganisms [18,24,25]. Both electrostatic and hydrophobic interactions induce bacterial membrane destabilization, lead to the disruption of the bilayer integrity or affect the membrane fluidity [26,27]. Moreover, the peptide could enter the cytoplasmic environment to interact with other potential bacterial targets. Although different models have been identified for cationic AMPs (barrel-stave, carpet, toroidal-tole and translocation) [25], the *Hv*RNASET2 mechanism of action could appear very similar. However, further experiments will be performed to better investigate the *Hv*RNASET2 mechanism of action and its ability to affect membranes. Since both Gram-positive and Gram-negative bacteria share the same cytoplasmic membrane, *Hv*RNASET2 could act against microorganisms regardless of their cell wall organization. Furthermore, Gram-negative bacteria display an additional potential target that is the outer membrane. Of note, other ribonucleases have been classified as antimicrobial proteins or enzymes, constituting a key first line of host defense against invading pathogens. The human RNase 3 (ECP), specifically secreted from the secondary granules of eosinophils, is released during inflammatory processes. ECP shows an antibacterial role independent of its catalytic activity, mediated by a cationic peptide in the N-terminal region that is able to induce both Gram-positive and Gram-negative bacteria agglutination and to inhibit cell growth [28].

Here, by means of optical and electron microscopy, a marked agglutinating effect on bacterial cells has been demonstrated for *Hv*RNASET2 as well. As previously reported in *S. aureus* [10], morphological studies performed on *E. coli* show that these microorganisms displayed a severe agglutination following *Hv*RNASET2 incubation compared to control samples. In particular, the aggregation process was clearly detectable by both TEM and SEM analyses, whereby not only were numerous cell clumps observed, but microbial membranes appeared significantl damaged, with the cytoplasmatic content released in the surrounding environment. Particularly after 24 and 48 h from *Hv*RNASET2 incubation, the leech recombinant enzyme destabilized outer membranes and affected their integrity. These effects are likely due to direct interaction with bacterial components, as confirmed by both immunogold at TEM and LPS binding assays. Of note, the presence of gold particles on the cell external surfaces at TEM indicates a direct interaction of HvRNASET2 with the bacterial cell wall. In parallel, the increase in the Body-P cadaverine fluorescent signal suggests the direct binding of leech T2 enzyme to LPS. It is tempting to speculate that such interaction could be mediated by the observed C-terminal peptide within *Hv*RNASET2, which might confer a bactericidal role to the enzyme. The administration of *Hv*RNASET2 to *E. coli* impaired in a significant manner the detection of viable cells. The observed decrease could be ascribable to a bactericidal effect and/or to an agglutination effect, as already observed in vivo experiments. Indeed, in silico analysis highlighted a putative antimicrobial stretch in RNASET2 that could affect cellular integrity. Moreover, an agglutination effect that can not be ruled out in the in vitro setup could underestimate the cellular concentration. However, regardless of the mechanism(s) involved, a statistically significant decrease of viable cells was observed upon HvRNASET2 treatment. 

Finally, in vivo experiments, in which bacterial cells were directly injected in the leech body wall, pointed out cell agglutination as a prerequisite for enhancing the host’s innate immune response. As already hypothesized [9], HvRNASET2 is specifically expressed and secreted by leech granulocytes during bacterial infections, playing a double role of recruiting host macrophages and at the same time interacting with bacterial pathogens. Indeed, the adhesion induced by rHvRNASET2 on *E. coli* living cells promotes the activation of phagocytic macrophages, as observed with ACP assay. Interestingly, this specific mechanism is similar to that observed in several C-type lectins detected in shrimps of the genus Penaeus. Indeed, the PmLec lectin of Penaeus mondon not only agglutinates *E. coli* but also binds to LPS in order to opsonize microbes and promote phagocytic events [29]. Moreover, in the larvae of the insect Manduca sexta, immunolectin-2 shows a high affinity for LPS and triggers the formation of bacterial cell aggregates, also inducing phenol oxidase defensive reaction [30]. Several cases of invertebrate immune molecules are reported in the literature that share the same effects, suggesting a common defensive mechanism being maintained throughout evolution in different organisms.

The discovery and the analysis of novel antimicrobial compounds will increase our knowledge of intrinsic host defenses and immune system. Identifying new alternative approaches to the expensive and poorly effective ones currently used is considered a key goal to improve human health. Exploiting the key features of the antimicrobial peptides such as efficiency and selectivity, broad range of targets, potentially low toxicity and accumulation in tissues, pharmaceutical industries aim to develop them as commercially available drugs, and several appropriate clinical trials are currently being conducted. Starting from our previous assumptions and experimental evidence [9,10,12], the present research aimed to shed light on leech *Hv*RNASET2, which displays several features that make it a potentially valuable tool to develop novel antibacterial molecules, also due to its wide-range ability to act on different types of microbial pathogens. 

## 4. Materials and Methods 

### 4.1. Bioinformatic Prediction of the Leech HvRNASET2 Characteristics 

The FASTA format of the *Hirudo verbana Hv*RNASET2 amino acid sequence (accession number: AYQ58237.1) was obtained from the National Center for Biotechnology Information (NCBI) (https://www.ncbi.nlm.nih.gov) GenBank database in order to conduct bioinformatic analyses. The *Hv*RNASET2 molecular weight (Mw) was calculated by ExPASy Compute pl/Mw Tool (https://web.expasy.org/compute_pi/), and both the amino acid length and the presence of active sites were determined using PROSITE server (https://prosite.expasy.org/scanprosite/). The N-terminal signal peptide was predicted using SignalP-5.0 program (http://www.cbs.dtu.dk/services/SignalP/) [31], and the TMHMM server (http://www.cbs.dtu.dk/services/TMHMM/) [32] was applied for determining the cellular enzyme localization.

### 4.2. Three-Dimensional Modelling of HvRNASET2

The three-dimensional structure of *Hv*RNASET2 was predicted with the I-TASSER (Iterative Threading ASSEmbly Refinement) server (https://zhanglab.ccmb.med.umich.edu/I-TASSER/) [33] based on the sequence homology of different structural templates present in the Protein Data Bank (PBD) library (https://www.rcsb.org/) [34]. The resulting PDB file was visualized and analyzed with PyMOL software (https://pymol.org/2/) in order to characterize the secondary structure of the enzyme.

### 4.3. HvRNASET2 Alignment and Conservation Analysis

Possible amino acid similarities and the relative conserved domains related to the T2 ribonuclease family were determined by the structural alignment between *Hv*RNASET2 and other members of the T2 RNase family by using Clustal Omega (https://www.ebi.ac.uk/Tools/msa/clustalo/) [35]. Two different multiple alignments were performed with the following T2 RNase enzymes: human RNASET2 (AIC50165.1); *Aspergillus niger* ACTIBIND (AAZ22530.1); *Hydra vulgaris* (XP_002164769.1); *Crassostrea gigas* (XP_011413920.2); *Temnothorax curvispinosus* (XP_024871065.1). The graphical output showed in the figure was viewed with the Jalview program (http://www.jalview.org/) [36] and manually created. 

### 4.4. Antimicrobial Peptide Identification

Based previous experimental evidence [10], the possible presence of an antimicrobial peptide was evaluated with AMPA software (http://tcoffee.crg.cat/apps/ampa/do) [13]. 

### 4.5. Light and Transmission Electron Microscopy

*E. coli* bacterial culture was grown at 37 °C overnight until reaching an OD600 of 0.6. Bacterial cells were then resuspended with the recombinant r*Hv*RNASET2 (10 µM) for 3, 24 and 48 h at 20 °C in order to conduct in vitro experiments. As control, microorganisms were treated with PBS solution (PBS: 138 mM NaCl, 2.7 mM KCl, 4.3 mM Na_2_HPO_4_ and 1.5 mM KH_2_PO_4_, pH 7.4) at the same times. After treatments, bacteria were centrifuged for 10 min at 13.000 rpm and fixed for 2 h in 0.1 M cacodylate buffer at pH 7.4, containing 2% glutaraldehyde. Several washings were performed in the same buffer, and then cells were postfixed for 1 h with 1% osmium tetroxide in cacodylate buffer, pH 7.4. Subsequently, samples were embedded in an Epon-Araldite 812 mixture (Sigma-Aldrich, Milan, Italy) after serial ethanol dehydration (70%, 90%, 100%). Sections were obtained with a Reichert Ultracut S ultratome (Leica, Wien, Austria). Semi-thin sections (0.7 μm) were colored by conventional methods, crystal violet and basic fuchsin and observed under a light microscope Nikon Eclipse Ni (Nikon, Tokyo, Japan). Data were recorded with a DS-5M-L1 digital camera system (Nikon). Ultrathin sections (80 nm) were collected on copper grids (300 mesh, Sigma-Aldrich, Milan, Italy), counterstained by uranyl acetate and lead citrate, and observed with a Jeol 1010 EX transmission electron microscope TEM (Jeol, Tokyo, Japan). Data were recorded with a MORADA digital camera system (Olympus, Tokyo, Japan).

### 4.6. Scanning Electron Microscopy

Reaching an OD600 of 0.6, *E. coli* bacteria were incubated as previously described. After treatments, cells were centrifuged for 10 min at 13,000 rpm, and bacterial pellets were fixed with Karnovsky fixative (2% paraformaldehyde and 2.5% glutaraldehyde in 0.1 M cacodylate Buffer, pH 7.2) for 30 min at 4 °C. Samples were washed in 0.1 M cacodylate buffer (pH 7.2) and post-fixed in a solution of 1% osmium tetroxide and potassium ferrocyanide for 1 h. After several washings in PBS (pH 7.2) and dehydration with an increasing scale of ethanol, 20 µL of bacterial pellet resuspended in 100% ethanol were dried onto glass slides and finally subjected to critical point drying with hexamethyldisilazane. Images were acquired using SEM-FEG XL-30 microscope (Philips, Eindhoven, The Netherlands).

### 4.7. Immunogold Staining at TEM

Bacterial samples were fixed for 2 h at 4 °C with 4% paraformaldehyde and 0.5% glutaraldehyde in PBS, dehydrated in ethanol series and embedded in an Epon-Araldite 812 mixture (Sigma-Aldrich) as above. Ultrathin sections (80 nm) were collected on gold grids (300 mesh, Sigma-Aldrich). After etching with NaOH 3% in absolute ethanol [37], slides were incubated for 30 min in blocking solution containing PBS, 1% bovine serum album (BSA) and 0.1% Tween and then with the polyclonal primary antibody rabbit anti-human RNASET2 [38] diluted at 1:20 in blocking solution. After several washings with PBS, the primary antibody was visualized by immunostaining, with the secondary goat anti-rabbit IgG(H+L)-gold conjugate antibody (GE Healthcare, Amersham, UK; particle size, 10 nm) diluted at 1:100 in blocking solution for 1 h. In control experiments, the primary antibody was omitted, and sections were treated with BSA containing PBS and incubated only with the secondary antibodies. Sections were counterstained with uranyl acetate in water and observed withTEM, and data were recorded with a digital camera system as previously described.

### 4.8. LPS Binding Assay

The binding with the Gram-negative antigen LPS was evaluated by a fluorescent displacement assay using the fluorescent probe Bodipy cadaverine (Thermo Fisher Scientific, Waltham, MA, USA). The dissociation constant was measured by adding small volumes of concentrated stock of both Polymixin B (Thermo Fisher Scientific, Waltham, MA, USA), used as positive control, and *Hv*RNASET2 to a solution of LPS (10 µM/mL) and fluorescent Bodipy TR cadaverine (10 µM) in 150 mM sodium phosphate buffer, pH 7.5, at 25 °C. Fluorescent Bodipy TR cadaverine normally binds to LPS, and the addition of a compound with a higher affinity for the bacterial antigen induces the detachment between Bodipy TR cadaverine and LPS and the consequent increase of the fluorescent signal. The fluorescence measurements were carried out with a 1 mL cell in a Jasco FP-750 spectrofluorometer (Jasco, Cremella, Italy) using an excitation wavelength of 580 nm. The change in emission at 620 nm was plotted as a function of ligand concentration.

### 4.9. Antimicrobial In Vitro Assay

*E. coli* BL21(DE3) was grown in LB broth at 37 °C under agitation. Upon overnight growth, cultures were diluted in fresh medium to reach a cellular concentration of ~10^5^ cfu/mL. A sample was treated with r*Hv*RNASET2 at a final concentration of 10 µM, and an untreated sample was included as control. Upon 24 h incubation, bacterial viability was checked by plate count technique and expressed as colony-forming units per mL (CFU/mL). A volume (0.01 mL) of undiluted or serially diluted samples was plated onto LB agar plates and incubated for 24 h at 37 °C. Experiments were independently repeated at least three times.

### 4.10. In Vivo Experiments: Animals and Treatments

Adult leeches (*Hirudo verbana*, Annelida, Hirudinea, from Ricarimpex, Eysines, France), measuring 10 cm were kept in lightly salted water (NaCl 1.5 g/mL), in aerated tanks and kept in an incubator at 20 °C. Before injection and/or dissection, leeches were anesthetized with a 10% ethanol solution, and all treatments were performed at the 80th superficial metamere from the oral sucker. Animals were randomly divided into separate experimental groups (three individuals for each time point) and submitted to various protocols and treatments, as described below:

Group 1: animals were injected with 50 μL of *E. coli* BL21(DE3) (10^8^ CFU/mL), grown overnight in LB liquid soil (LB Broth, Sigma Aldrich, Milan, Italy). Bacteria, at an OD600 of 0.6, were injected after 3, 24 and 48 h of incubation, and leech tissues were collected 3 h after stimulation.

Group 2: animals were injected with 50 μL of *E. coli* BL21(DE3) (10^8^ CFU/mL), grown overnight in LB liquid soil, resuspended in a 10 μM recombinant *Hv*RNASET2 enzyme solution. Bacteria, at an OD600 of 0.6, were injected after 3, 24 and 48 h of incubation, and leech tissues were collected after 3 h from stimulation.

Group 3: animals were injected with 50 μL of *E. coli* BL21(DE3) (10^8^ CFU/mL), grown overnight in LB liquid soil, resuspended in sterilized phosphate buffer saline (PBS). Bacteria, at an OD600 of 0.6, were injected after 3, 24 and 48 h of incubation, and leech tissues were collected after 3 h of stimulation. 

### 4.11. Light Microscopy and Acid Phosphatase Assay (ACP)

Leech tissue samples, dissected from differently treated leech body walls, were embedded in Polyfreeze tissue freezing medium (OCT, Polysciences, Eppelheim, Germany) and immediately frozen in liquid nitrogen. Cryosections (7 µm) were obtained with a cryotome (Leica CM1850, Wetzlar, Germany), collected on gelatinous slides and counterstained with crystal violet and basic fuchsin for morphological analysis. All samples were mounted with Cityfluor (Cityfluor Ltd., London, UK) and examined with a Nikon Eclipse Ni (Nikon, Tokyo, Japan) light microscope. Data were recorded with a Nikon digital sight DS-SM (Nikon). 

For ACP assay, cryosections were rehydrated with PBS and incubated with sodium acetate-acetic acid 0.1 M buffer for 5 min, followed by incubation with reaction mixture (sodium acetate-acetic acid 0.1 M buffer, 0.01% naphtol ASBI phosphate, 2% NN-dimethylformamide, 0.06% Fast RedViolet LB and MnCl_2_ 0.5 nM) for 90 min at 37 °C. After several washings in PBS, slides were mounted with PBS/glycerol and observed with a Nikon Eclipse Ni (Nikon) as above. 

### 4.12. Statistical Analyses

Statistical analyses were conducted on AMP and ACP assays. All the experiments were organized in triplicate, and data represented the mean values ± SD. In detail, for both experiments, either the total number of bacterial colonies or positive phagocytic macrophages were counted by hand. For the ACP assay, activated cells were assessed by analyzing five different slides (random fields of 45,000 μm^2^ for each slide) for each experiment, using the ImageJ software package. Subsequently, statistical analyses were executed using Statistica 7.0 software (StatSoft Inc., Tulsa, OK, USA), and differences were calculated respectively by unpaired t-test and one-way ANOVA followed by Tukey’s post-hoc test. *p* < 0.05 was considered statistically significant. The related graphs were created with GraphPad Prism 7 (GraphPad Software, La Jolla, CA, USA).

## Supplementary Materials

Supplementary Materials can be found at https://www.mdpi.com/1422-0067/21/24/9722/s1. Figure S1. Bioinformatic insight.

## Abbreviations

AMPs	Antimicrobial peptides
LTA	Lipoteichoic acid
LPS	Lipopolysaccharide
SEM	Scanning electron microscopy
TEM	Transmission electron microscopy
PDB	Protein Data Bank
ACP	Acid phosphatase assay

## References

[1] Sirijan S. (2016). Nitaya Indrawattana Mechanisms of antimicrobial resistance in Pasteurellaceae. BioMed Res. Int..

[2] Mukhopadhyay S., Bharath Prasad A.S., Mehta C.H., Nayak U.Y. (2020). Antimicrobial peptide polymers: No escape to ESKAPE pathogens—A review. World J. Microbiol. Biotechnol..

[3] Mahlapuu M., Björn C., Ekblom J. (2020). Antimicrobial peptides as therapeutic agents: Opportunities and challenges. Crit. Rev. Biotechnol..

[4] Irie M. (1999). Structure-Function Relationships of Acid Ribonucleases. Pharmacol. Ther..

[5] Deshpande R.A., Shankar V. (2002). Ribonucleases from T2 family. Crit. Rev. Microbiol..

[6] Luhtala N., Parker R. (2010). T2 Family ribonucleases: Ancient enzymes with diverse roles. Trends Biochem. Sci..

[7] Acquati F., Bertilaccio S., Grimaldi A., Monti L., Cinquetti R., Bonetti P., Lualdi M., Vidalino L., Fabbri M., Sacco M.G. (2011). Microenvironmental control of malignancy exerted by RNASET2, a widely conserved extracellular RNase. Proc. Natl. Acad. Sci. USA.

[8] Acquati F., Lualdi M., Bertilaccio S., Monti L., Turconi G., Fabbri M., Grimaldi A., Anselmo A., Inforzato A., Collotta A. (2013). Loss of function of Ribonuclease T2, an ancient and phylogenetically conserved RNase, plays a crucial role in ovarian tumorigenesis. Proc. Natl. Acad. Sci. USA.

[9] Baranzini N., Monti L., Vanotti M., Orlandi V.T., Bolognese F., Scaldaferri D., Girardello R., Tettamanti G., De Eguileor M., Vizioli J. (2019). AIF-1 and RNASET2 Play Complementary Roles in the Innate Immune Response of Medicinal Leech. J. Innate Immun..

[10] Baranzini N., De Vito A., Orlandi V.T., Reguzzoni M., Monti L., de Eguileor M., Rosini E., Pollegioni L., Tettamanti G., Acquati F. (2020). Antimicrobial Role of RNASET2 Protein during Innate Immune Response in the Medicinal Leech Hirudo verbana. Front. Immunol..

[11] de Eguileor M., Grimaldi A., Tettamanti G., Valvassori R., Cooper E.L., Lanzavecchia G. (2000). Different types of response to foreign antigens by leech leukocytes. Tissue Cell.

[12] Baranzini N., Pedrini E., Girardello R., Tettamanti G., de Eguileor M., Taramelli R., Acquati F., Grimaldi A. (2017). Human recombinant RNASET2-induced inflammatory response and connective tissue remodeling in the medicinal leech. Cell Tissue Res..

[13] Torrent M., Di Tommaso P., Pulido D., Nogués M.V., Notredame C., Boix E., Andreu D. (2012). AMPA: An automated web server for prediction of protein antimicrobial regions. Bioinformatics.

[14] Baranzini N., Weiss-Gayet M., Chazaud B., Monti L., de Eguileor M., Tettamanti G., Acquati F., Grimaldi A. (2020). Recombinant HvRNASET2 protein induces marked connective tissue remodelling in the invertebrate model Hirudo verbana. Cell Tissue Res..

[15] Grafskaia E.N., Nadezhdin K.D., Talyzina I.A., Polina N.F., Podgorny O.V., Pavlova E.R., Bashkirov P.V., Kharlampieva D.D., Bobrovsky P.A., Latsis I.A. (2019). Medicinal leech antimicrobial peptides lacking toxicity represent a promising alternative strategy to combat antibiotic-resistant pathogens. Eur. J. Med. Chem..

[16] Torrent M., Navarro S., Moussaoui M., Nogués M.V., Boix E. (2008). Eosinophil Cationic Protein High-Affinity Binding to Bacteria-Wall Lipopolysaccharides and Peptidoglycans. Biochemistry.

[17] Ageitos J.M., Sánchez-Pérez A., Calo-Mata P., Villa T.G. (2017). Antimicrobial peptides (AMPs): Ancient compounds that represent novel weapons in the fight against bacteria. Biochem. Pharmacol..

[18] Silva J.P., Appelberg R., Gama F.M. (2016). Antimicrobial peptides as novel anti-tuberculosis therapeutics. Biotechnol. Adv..

[19] Campomenosi P., Salis S., Lindqvist C., Mariani D., Nordström T., Acquati F., Taramelli R. (2006). Characterization of RNASET2, the first human member of the Rh/T2/S family of glycoproteins. Arch. Biochem. Biophys..

[20] Acquati F., Possati L., Ferrante L., Campomenosi P., Talevi S., Bardelli S., Margiotta C., Russo A., Bortoletto E., Rocchetti R. (2005). Tumor and metastasis suppression by the human RNASET2 gene. Int. J. Oncol..

[21] Thorn A., Steinfeld R., Ziegenbein M., Grapp M., Hsiao H.-H., Urlaub H., Sheldrick G.M., Gärtner J., Krätzner R. (2012). Structure and activity of the only human RNase T2. Nucleic Acids Res..

[22] Kawata Y., Sakiyama F., Hayashi F., Kyogoku Y. (1990). Identification of two essential histidine residues of ribonuclease T2. Eur. J. Biochem..

[23] Irie M. (1997). RNase T1/RNase T2 Family RNases. Ribonucleases.

[24] Malmsten M. (2015). Interactions of Antimicrobial Peptides with Bacterial Membranes and Membrane Components. Curr. Top. Med. Chem..

[25] Geitani R., Moubareck C.A., Xu Z., Karam Sarkis D., Touqui L. (2020). Expression and Roles of Antimicrobial Peptides in Innate Defense of Airway Mucosa: Potential Implication in Cystic Fibrosis. Front. Immunol..

[26] Sharma S., Sahoo N., Bhunia A. (2015). Antimicrobial Peptides and their Pore/Ion Channel Properties in Neutralization of Pathogenic Microbes. Curr. Top. Med. Chem..

[27] Omardien S., Drijfhout J.W., Vaz F.M., Wenzel M., Hamoen L.W., Zaat S.A.J., Brul S. (2018). Bactericidal activity of amphipathic cationic antimicrobial peptides involves altering the membrane fluidity when interacting with the phospholipid bilayer. Biochim. Biophys. Acta Biomembr..

[28] Torrent M., Sánchez D., Buzón V., Nogués M.V., Cladera J., Boix E. (2009). Comparison of the membrane interaction mechanism of two antimicrobial RNases: RNase 3/ECP and RNase 7. Biochim. Biophys. Acta Biomembr..

[29] Luo T., Yang H., Li F., Zhang X., Xu X. (2006). Purification, characterization and cDNA cloning of a novel lipopolysaccharide-binding lectin from the shrimp Penaeus monodon. Dev. Comp. Immunol..

[30] Yu X.Q., Kanost M.R. (2000). Immulectin-2, a lipopolysaccharide-specific lectin from an insect, Manduca sexta, is induced in response to Gram-negative bacteria. J. Biol. Chem..

[31] Almagro Armenteros J.J., Tsirigos K.D., Sønderby C.K., Petersen T.N., Winther O., Brunak S., von Heijne G., Nielsen H. (2019). SignalP 5.0 improves signal peptide predictions using deep neural networks. Nat. Biotechnol..

[32] Möller S., Croning M.D.R., Apweiler R. (2001). Evaluation of methods for the prediction of membrane spanning regions. Bioinformatics.

[33] Zhang Y. (2008). I-TASSER server for protein 3D structure prediction. BMC Bioinform..

[34] Berman H.M., Battistuz T., Bhat T.N., Bluhm W.F., Bourne P.E., Burkhardt K., Feng Z., Gilliland G.L., Iype L., Jain S. (2002). The protein data bank. Acta Crystallogr. Sect. D Biol. Crystallogr..

[35] Sievers F., Wilm A., Dineen D., Gibson T.J., Karplus K., Li W., Lopez R., McWilliam H., Remmert M., Söding J. (2011). Fast, scalable generation of high-quality protein multiple sequence alignments using Clustal Omega. Mol. Syst. Biol..

[36] Waterhouse A.M., Procter J.B., Martin D.M.A., Clamp M., Barton G.J. (2009). Jalview Version 2-A multiple sequence alignment editor and analysis workbench. Bioinformatics.

[37] Causton B.E. (1984). The Choice of Resins for Electron Immunocytochemistry.

[38] Campomenosi P., Cinquetti R., Tallarita E., Lindqvist C., Raimondi I., Grassi P., Näsman J., Dell A., Haslam S.M., Taramelli R. (2011). Comparison of the baculovirus-insect cell and Pichia pastoris heterologous systems for the expression of the human tumor suppressor protein RNASET2. Biotechnol. Appl. Biochem..

