# Supplementary material for: 3D Reconstruction of HvRNASET2 Molecule to Understand Its Antibacterial Role
Source: Int J Mol Sci. 2020 Dec 19;21(24):9722. doi: 10.3390/ijms21249722 (PMC7767213; doi:10.3390/ijms21249722)
Supplement: Supplementary file 1 [file ijms-21-09722-s001.pdf]

Supplementary Figure

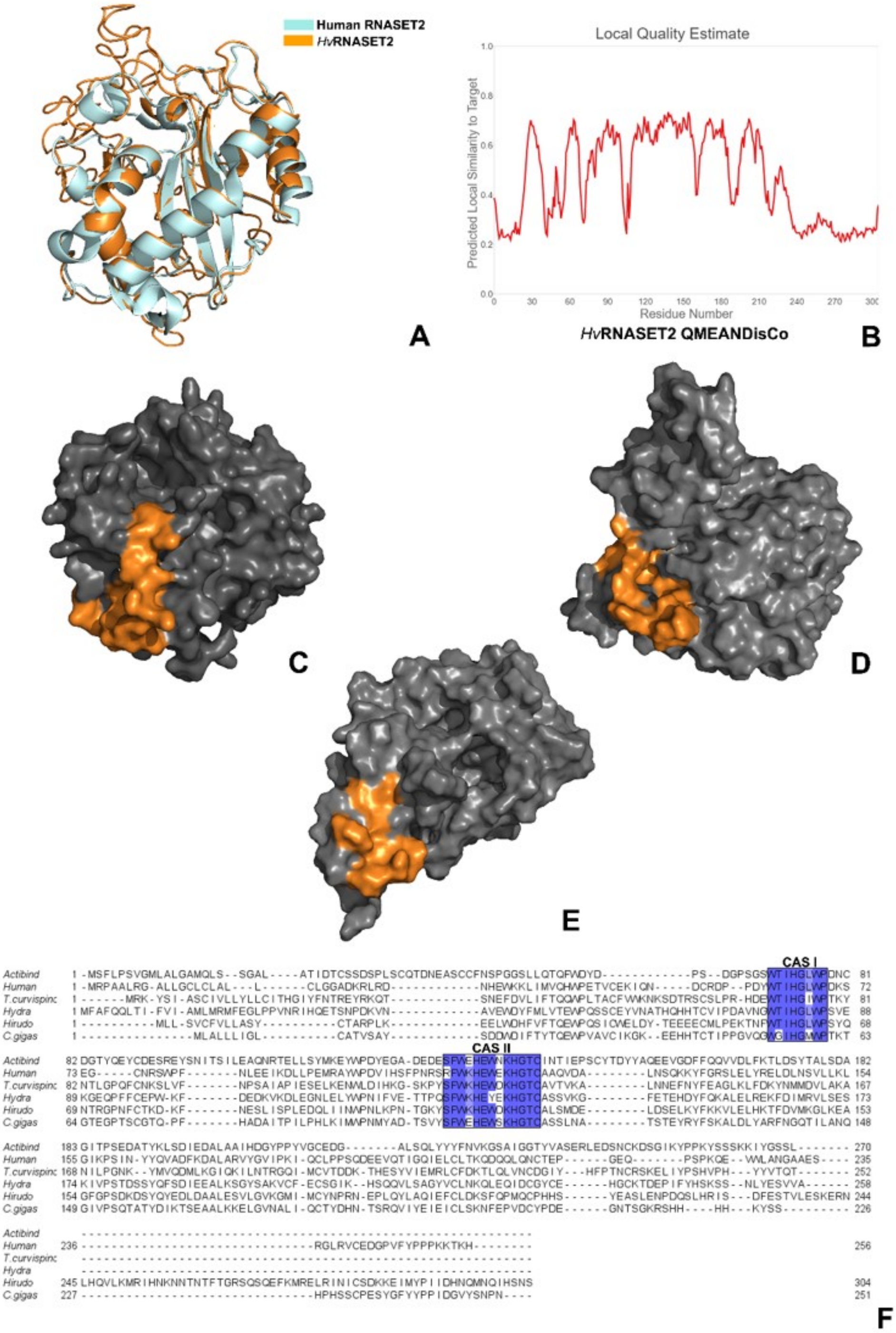

Figure S1. (**A**) Three-dimensional alignment of human RNASET2 (in cyan) and H. verbana HvRNASET2 (in orange). The secondary structures (β-strands and α-helix) appear extremely conserved. (**B)** Graphical representation of the HvRNASET2 QMEANDisCo score. The plot shows the precise QMean value of every amino acid. (**C-E**) Alternative three-dimensional models of HvRNASET2 obtained with the following servers for 3D structures prediction: Phyre2 (**C**), Robetta-Baker Lab (**D**) and RaptorX Contact Predict (**E**). Of note, the antimicrobial peptide (QVLKMRIHNKNNTNTFT), represented in orange, is specifically localized on the external surface of the enzyme. (**F**) Multiple sequence alignments. The HvRNASET2 amino acid sequence has been

compared with all the sequences considered in the Figure 1. The complete alignment confirms that the conserved active sites (CAS I and CAS II) are extremely preserved. Different blue shades identify the diverse conservation of each amino acid.
